# Controlled Release of Collagen-Binding SDF-1α Improves Cardiac Function after Myocardial Infarction by Recruiting Endogenous Stem Cells

**DOI:** 10.1038/srep26683

**Published:** 2016-05-26

**Authors:** Jie Sun, Yannan Zhao, Qingguo Li, Bing Chen, Xianglin Hou, Zhifeng Xiao, Jianwu Dai

**Affiliations:** 1 Institute of Combined Injury, State Key Laboratory of Trauma, Burns and Combined Injury, Chongqing Engineering Research Center for Nanomedicine, College of Preventive Medicine, Third Military Medical University, Chongqing 400038, China; 2 State Key Laboratory of Molecular Developmental Biology, Institute of Genetics and Developmental Biology, Chinese Academy of Sciences, Beijing 100080, China; 3 Department of Cardiothoracic Surgery, the Second Affiliated Hospital of Nanjing Medical University, Nanjing, China

## Abstract

Stromal cell-derived factor-1α (SDF-1α) is a well-characterized chemokine that mobilizes stem cells homing to the ischemic heart, which is beneficial for cardiac regeneration. However, clinically administered native SDF-1α diffuses quickly, thus decreasing its local concentration, and results in side effects. Thus, a controlled release system for SDF-1α is required to produce an effective local concentration in the ischemic heart. In this study, we developed a recombinant chemokine, consisting of SDF-1α and a collagen-binding domain, which retains both the SDF-1α and collagen-binding activity (CBD-SDF-1α). In an *in vitro* assay, CBD-SDF-1α could specifically bind to a collagen gel and achieve sustained release. An intramyocardial injection of CBD-SDF-1α after acute myocardial infarction demonstrated that the protein was largely tethered in the ischemic area and that controlled release had been achieved. Furthermore, CBD-SDF-1α enhanced the recruitment of c-kit positive (c-kit^+^) stem cells, increased capillary density and improved cardiac function, whereas NAT-SDF-1α had no such beneficial effects. Our findings demonstrate that CBD-SDF-1α can specifically bind to collagen and achieve controlled release both *in vitro* and *in vivo*. Local delivery of this protein could mobilize endogenous stem cells homing to the ischemic heart and improve cardiac function after myocardial infarction.

Myocardial infarction (MI) is the principal cause of mortality throughout the world and leads to cardiomyocyte loss, scar formation and ventricular remodeling. Angioplasty and bypass surgery are the most common approaches to restore blood supply to the ischemic myocardium, but they can only postpone the disease progression and do not induce myocardial regeneration. Stem cell-based therapy is a promising strategy for myocardial repair that can improve cardiac function via secreting growth factors and replenishing dysfunctional cells^1^,^2^. However, direct injection of stem cells into the ischemic hearts has failed to improve cardiac function because the microenvironment of the ischemic myocardium does not promote exogenous cells survival, differentiation and integration into the recipient heart. A series of other problems, including immune rejection and infection, also pose challenges. Recently, therapeutic strategies focused on mobilizing endogenous stem cells homing to the ischemic heart have produced promising results for cardiac regeneration. Previous studies have suggested that endogenous stem cells from the systemic circulation or the surrounding heart could serve as a vital source for cardiac repair after myocardial infarction^3^,^4^,^5^. Endogenous stem cells homing to the injured sites might contribute to angiogenesis by secreting angiogenic factors and directly participating in microvessel formation or replenishing cardiomyocytes^6^,^7^. Thus, this behavior holds promise for a new strategy involving harnessing the heart’s intrinsic regenerative potential without delivering exogenous stem cells.

The chemokine SDF-1α is a novel candidate to mobilize endogenous stem cells homing to the ischemic heart^6^,^9^,^10^. The expression of SDF-1α is upregulated after acute myocardial infarction (AMI). SDF-1α can mobilize stem cells homing to the ischemic heart, increase capillary density and improve cardiac function, but it maintains a high concentration for only a short period^11^,^12^,^13^.

Many studies have attempted to increase the SDF-1α concentration in the ischemic area by transplanting SDF-1α-overexpressing cells or using multiple injections, which have been shown to increase the stem cell infiltration^14^,^15^,^16^. Although these beneficial effects of SDF-1α are well known, native SDF-1α diffuses quickly *in vivo*. A high dose of SDF-1α would increase the treatment cost and produce undesirable adverse effects. Therefore, it is important to identify a suitable delivery system for the sustained release of SDF-1α to mobilize endogenous stem cells homing to desirable sites.

Expression of type I collagen, the main component of the cardiac extracellular matrix, increases after AMI^17^. Thus, we hypothesize that collagen may be a potential target for the controlled release of SDF-1α, which may enhance the local concentration of SDF-1α and more effectively recruit endogenous stem cells. In a previous study, de Souza *et al.* have demonstrated that a heptapeptide (TKKTLRT) derived from the collagen-binding domain (CBD) of collagenase can specifically bind to collagen^18^. In our previous work, we produced a collagen-binding vascular endothelial growth factor (CBD-VEGF) by fusing the collagen-binding domain with VEGF, which has been shown to achieve controlled release and improve cardiac function in an acute myocardial infarction model^19^.

In the present study, we designed a fusion protein that consists of SDF-1α and the collagen-binding domain (CBD-SDF-1α) and that can specifically bind to collagen and achieve controlled release both *in vitro* and *in vivo*. In the rat acute myocardial infarction model, CBD-SDF-1α was retained and maintained a high concentration at the border zone of the ischemic area. This protein could also mobilize endogenous stem cells homing to the infarcted heart, increase the capillary density and improve cardiac function.

## Results

### CBD-SDF-1α can specifically bind to collagen and achieve controlled release

**Figure 1:**
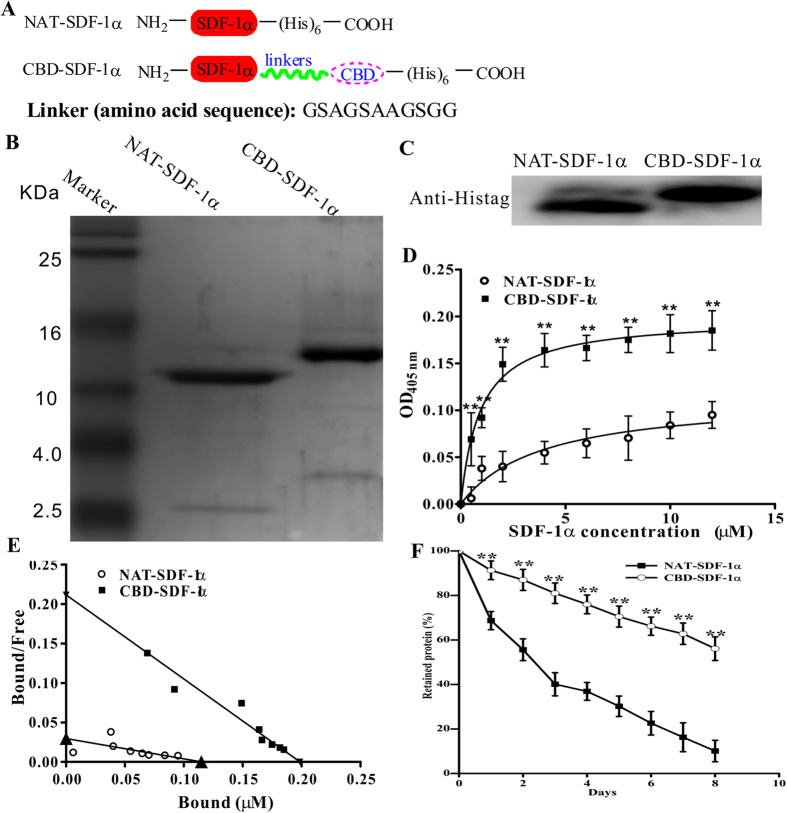
Characterization of NAT-SDF-1α and CBD-SDF-1α *in vitro*. (**A**) Schematic diagram of the construction of NAT-SDF-1α and CBD-SDF-1α. A collagen-binding domain was incorporated into the C-terminus of NAT-SDF-1α, and they were separated by a linker. (**B**) NAT-SDF-1α and CBD-SDF-1α were identified by Tricine-SDS-PAGE. (**C**) NAT-SDF-1α and CBD-SDF-1α were identified by western blotting using a monoclonal anti-polyhistidine antibody. (**D**) The binding curves of NAT-SDF-1α and CBD-SDF-1α to collagen were measured by ELISA. (**E**) Dissociation curves of NAT-SDF-1α and CBD-SDF-1α. (**F**) Controlled release curves of NAT-SDF-1α and CBD-SDF-1α from collagen. The data are presented as the means ± SEM, **P < 0.01.

To preserve the bioactivity of SDF-1α, the collagen-binding domain was fused to the C-terminus of native SDF-1α using a linker to separate the active domains (Fig. 1A). NAT-SDF-1α and CBD-SDF-1α were expressed in *E. coli*, and the purified proteins were identified by Tricine-SDS-PAGE (Fig. 1B) and western blotting (Fig. 1C). A modified ELISA assay was performed to measure the collagen-binding ability of CBD-SDF-1α *in vitro*. As shown in Fig. 1D, with concentrations ranging from 0.5–12 μM, the OD values of CBD-SDF-1α were significantly higher than those of NAT-SDF-1α (n = 6, *P* < 0.01) at same concentrations, which indicated that more CBD-SDF-1α bound to collagen. The *K*_d_ values of NAT-SDF-1α and CBD-SDF-1α were calculated by Scatchard analysis, and the slope of the resulting straight line equals −1/*K*_d_. As shown in Fig. 1E, the *K*_d_ values of NAT-SDF-1α and CBD-SDF-1α were calculated as 1.339 and 0.254 μM, respectively, and the lower *K*_d_ value of CBD-SDF-1α represented a higher affinity to collagen.

Next, the sustained releases of NAT-SDF-1α and CBD-SDF-1α were observed for up to 8 days. As shown Fig. 1F, a substantial amount of NAT-SDF-1α was quickly released on the first day, whereas CBD-SDF-1α was gradually released from collagen. At day 8, 56.1% of CBD-SDF-1α was retained on collagen, whereas only 10.1% of NAT-SDF-1α was retained (*P* < 0.01). All of the results indicated that CBD-SDF-1α could be controllably released from collagen.

### CBD-SDF-1α and NAT-SDF-1α have similar biological activity

**Figure 2:**
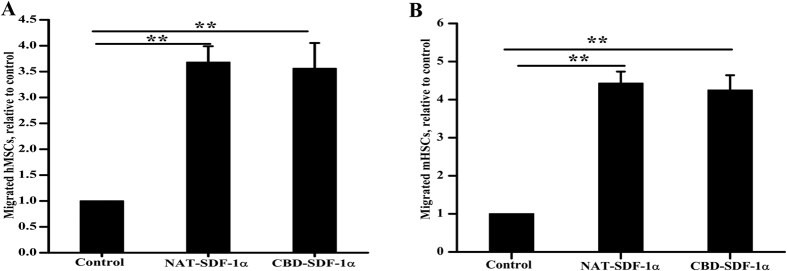
CBD-SDF-1α and NAT-SDF-1α exhibit similar activity in mediating hMSC and mHSC migration. (**A**) Chemokine-induced chemotaxis of hMSCs in a modified Boyden chamber. (**B**) Chemokine-induced chemotaxis of mHSCs. CBD-SDF-1α exhibits similar chemotactic activity as NAT-SDF-1α, which indicated that the fusion of a collagen-binding domain to SDF-1α did not affect its bioactivity. The data are presented as the means ± SEM, **P < 0.01.

CXCR4, the receptor for SDF-1α, is expressed on the membranes of many stem cells, including HSCs and MSCs. The bioactivities of NAT-SDF-1α and CBD-SDF-1α were also evaluated by inducing the migration of mHSCs and hMSCs. The data indicated that both of NAT-SDF-1α and CBD-SDF-1α (100 ng/mL) significantly mediated mHSC and hMSC migration compared to the control group (n = 6, *P* < 0.01). There was no significant difference between NAT-SDF-1α and CBD-SDF-1α (Fig. 2A,B), which demonstrated that the bioactivity of SDF-1α was not affected by its fusion with the CBD peptides at the C-terminus.

### The CBD-SDF-1α-modified collagen gel captured more mHSCs and hMSCs

**Figure 3:**
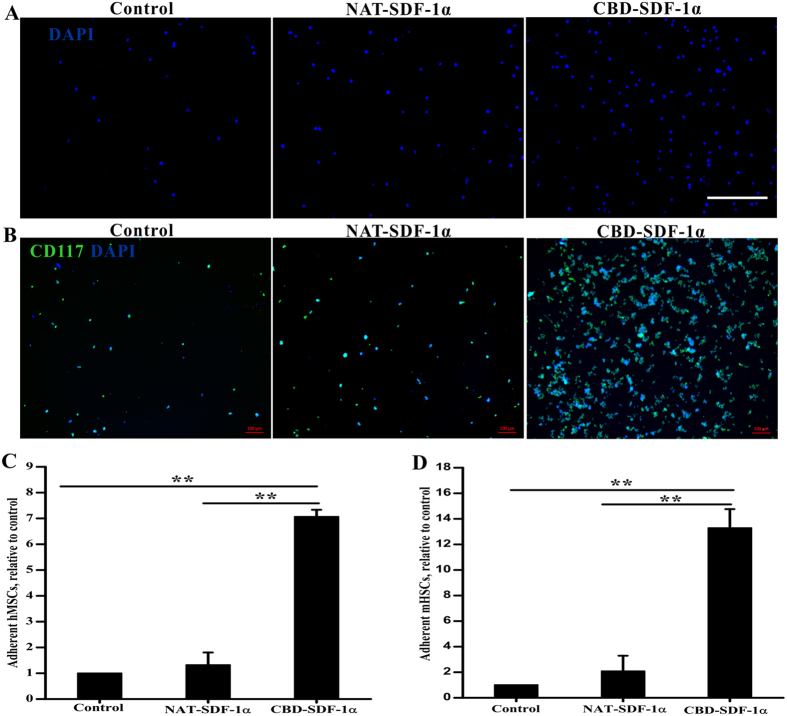
The adhesive ability of hMSCs and mHSCsis mediated by CBD-SDF-1α. Representative images of adhesive hMSCs (Blue-DAPI) (**A**) and mHSCs (Blue-DAPI, Green-CD117) (**B**) retained on the different collagen gels. (**C**) Quantitative analysis of the fold changes in the number of adherent hMSCs relative to the control group. (**D**) Quantitative analysis of the fold changes in the number of adherent mHSCs relative to the control group. Each assay was performed in triplicate. The data are presented as the means ± SEM, **P < 0.01. Scale bar = 100 μm.

To analyze whether CBD-SDF-1α could capture more stem cells via the ligand-receptor interaction, MSCs and HSCs were prepared and incubated on NAT-SDF-1α- or CBD-SDF-1α-modified collagen gels, respectively. MSC has been identified by flow cytometry, which are positive for CD73, CD90 and CD105, while negative for CD34, CD45 and CD106 (Fig. S1A). Moreover, these cells have uniform fibroblast-like morphology and can differentiate into osteoblasts, adipocytes and chondrocytes (Fig. S1B), indicating that these cells are MSCs. The co-staining images of CD117 and DAPI showed that mHSCs have good identity (Fig. 3B). As we expected, the CBD-SDF-1α-modified collagen gel increased the adhesion of hMSCs and mHSCs by 7.07-fold (Fig. 3A,C) and 13.29-fold (Fig. 3B,D), respectively, compared to the blank collagen gel (*P* < 0.01), whereas the collagen gel loaded with NAT-SDF-1α could not effectively capture the HSCs or MSCs. Because the NAT-SDF-1α has no collagen-binding ability, it quickly diffuses when loaded onto the collagen gel.

### CBD-SDF-1α is retained and exhibits controlled release in the ischemic area

**Figure 4:**
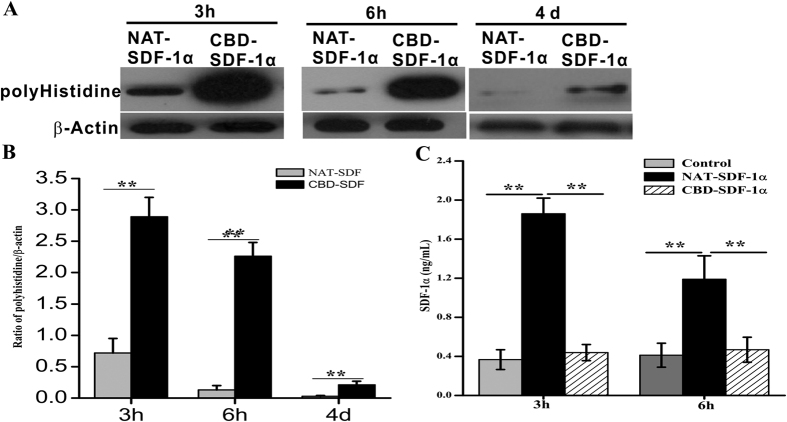
CBD-SDF-1α can bind to the ischemic area and achieve controlled release. **(A**) Western blotting was used to measure the exogenous SDF-1α in the ischemic area at 3 hours, 6 hours and 4 days after injection using an anti-polyhistidine antibody, and β-actin was used as the internal control. (**B**) Quantification of the protein bands (n = 6 in each group). (**C**) The SDF-1α levels in serum were measured at 3 and 6 hours after injection (n = 6 in each group). The data are presented as the means ± SEM, **P < 0.01.

To ascertain whether CBD-SDF-1α could bind to endogenous collagen, NAT-SDF-1α or CBD-SDF-1α was injected into the border zone of the ischemic sites. At each time point, the cardiac proteins were extracted for western blot analysis and an anti-polyhistidine antibody was used to distinguish the exogenous SDF-1α from the endogenous SDF-1α in the ischemic heart. As shown in Fig. 4A,B, NAT-SDF-1α could be detected at 3 hours but had almost completely disappeared at 6 hours after injection. In contrast, the CBD-SDF-1α levels decreased slowly and a large amount of this protein could be detected at 6 hours. At day 4 post-surgery, there was a small amount of CBD-SDF-1α could be detected. But at day 7 post-surgery, neither CBD-SDF-1α nor NAT-SDF-1α could be detected by western blot. These resulted indicated that CBD-SDF-1α could be retained in the ischemic area within one week, and the recruitment of endogenous stem cells should occur within one week after damage.

To demonstrate whether the NAT-SDF-1α or CBD-SDF-1α diffused into the peripheral blood, we measured the levels of SDF-1α in the serum at 3 and 6 hours after injection. As shown in Fig. 4C, the concentration of SDF-1α in the NAT-SDF-1α group was higher than in of the CBD-SDF-1α group. These data demonstrated that NAT-SDF-1α diffused quickly *in vivo*, whereas CBD-SDF-1α could bind to endogenous collagen, maintain a high concentration and achieve sustained release.

### CBD-SDF-1α promotes the recruitment of c-kit^+^ stem cells and the expression of VEGF in the ischemic area

**Figure 5:**
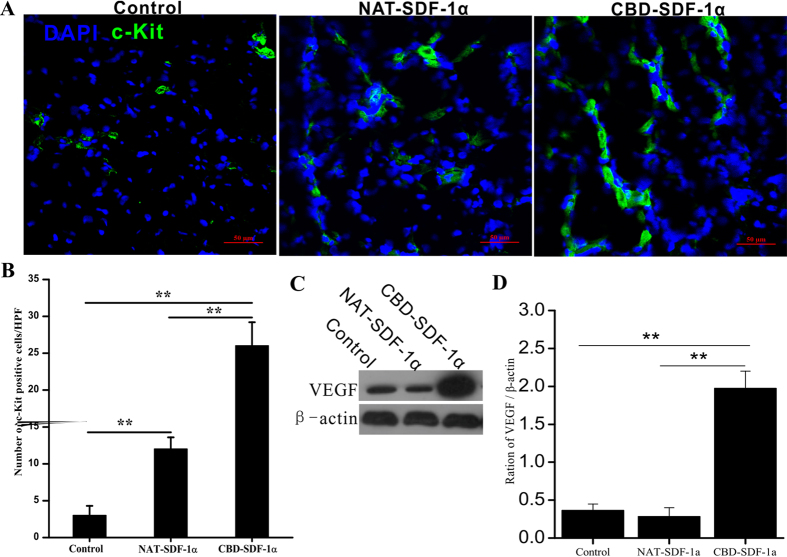
Recruitment of c-kit^+^ stem cells and the level of VEGF the ischemic area. (**A**) Representative images of the c-kit^+^ stem cells that migrated into the ischemic area at 4 days after surgery. (**B**) Average number of c-kit^+^ cells in the infarcted heart (n = 6 in each group). CBD-SDF-1α mobilized more c-kit^+^ cells to migrate to the infarcted heart. (**C**) The level of VEGF in the ischemic area was detected by Western blot. (**D**) Quantification of the protein bands (n = 5 in each group). The data are presented as the means ± SEM, **P < 0.01.

To demonstrate whether the controlled release of SDF-1α could enhance the recruitment of endogenous stem cells, we used c-kit as a marker to detect the stem cells at 4 days after AMI. As shown in Fig. 5A,B, local delivery of CBD-SDF-1α recruited more c-kit^+^ stem cells to the ischemic area than the control and NAT-SDF-1α groups (*P* < 0.01, respectively), and the recruited stem cells may play key roles in cardiac regeneration and repair. The results of western bolt showed that compared with control and NAT-SDF-1α groups, CBD-SDF-1α could elevate higher level of VEGF in the ischemic area, which is a key therapeutic factor in myocardial regeneration (Fig. 5C,D).

### CBD-SDF-1α improves cardiac function after MI

Cardiac function was evaluated by echocardiography at 12 weeks after myocardial infarction. As shown in Table 1, the LV fractional shortening and ejection fraction were significantly higher in the CBD-SDF-1α group than in the NAT-SDF-1α and control groups (*P* < 0.05). No significant difference was found in LVDd, LVDs, IVSDT, or LVPWT among the control, NAT-SDF-1α, and CBD- SDF-1αgroups.

### CBD-SDF-1α reduces the scar size and increases the LV wall thickness after MI

**Figure 6:**
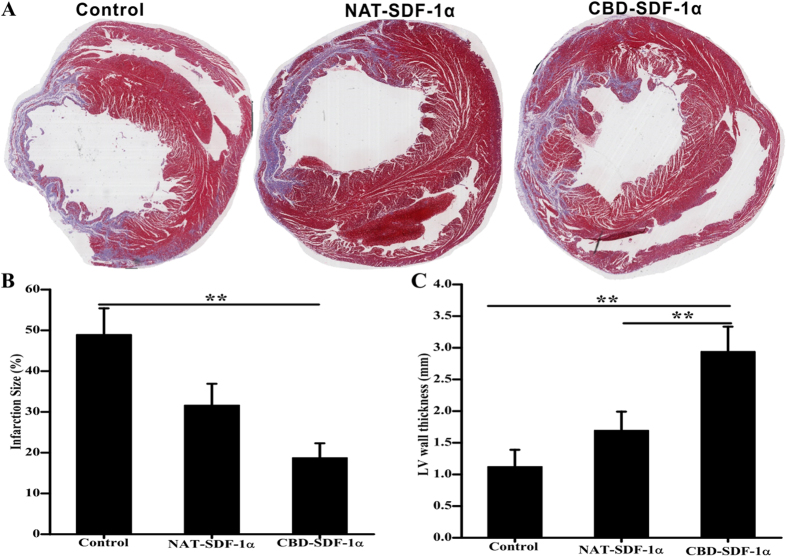
Measurement of infarct size and LV wall thickness. (**A**) Representative images of Masson’s trichrome staining. (**B**) The proportion of scar tissues (blue) in the left ventricle was determined by calculating the ratio of the area of scar to the left ventricular area (n = 8 in each group). (**C**) Average thickness of the LV wall in the infarcted region (n = 8 in each group). CBD-SDF-1α reduced the infarct size and increased the LV wall thickness. The data are presented as the means ± SEM, **P < 0.01.

Masson’s trichrome staining was performed to measure the scar size and LV wall thickness. As shown in Fig. 6, the percentage of the scar size in the CBD-SDF-1α group (18.7 ± 4.62%) was significantly reduced compared to the control (48.39 ± 5.68%) and NAT-SDF-1α (31.83 ± 6.75%) groups (*P* < 0.05). The protective effects were also reflected by the left ventricular wall thickness. The wall thickness in the CBD-SDF-1α group (2.73 ± 0.39 mm) was significantly greater than that in the NAT-SDF-1α (1.52 ± 0.39 mm) and control (1.25 ± 0.41 mm) groups (*P* < 0.01). The reduction in the scar size and increased LV wall thickness indicated that CBD-SDF-1α promotes cardiac regeneration and repair.

### CBD-SDF-1α promotes angiogenesis afterMI

**Figure 7:**
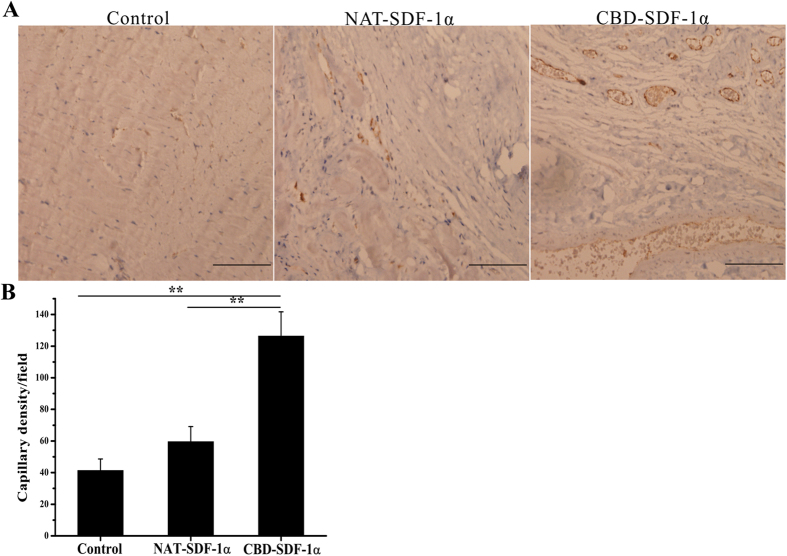
Histological analysis of capillary density in the ischemic area using antibody to vWF. (**A**) Representative images of vWF staining. (**B**) Average blood vessel density (n = 8 in each group). CBD-SDF-1α increased the capillary density in the ischemic area. The data are presented as the means ± SEM, **P < 0.01. Scale bar = 50 μm.

Although the above results demonstrated that CBD-SDF-1α can improve cardiac function and reduce scar size, the myocardium ultimately must be vascularized for long-term cardiomyocyte survival. Hence, we investigated the number of capillary vessels at 12 weeks after myocardial infarction by using an antibody to vWF, a well-known marker of the mature microvasculature. The images in Fig. 7 display a significantly higher capillary density at the border zone in the CBD-SDF-1α-treated hearts (275.61 ± 18.25/mm^2^) compared to the NAT-SDF-1α (183.28 ± 20.36/mm^2^) and control (161.49 ± 17.92/mm^2^) groups, demonstrating that CBD-SDF-1α could promote angiogenesis.

## Discussion

Mobilizing endogenous stem cells homing to the injured heart could promote cardiac repair and improve cardiac function. Many studies have demonstrated that SDF-1α could mobilize endogenous stem cells to migrate to the injured tissue and promote wound repair and regeneration. The chemokine SDF-1α and its receptor, CXCR4, have attracted considerable attention as a result of their involvement in mobilizing stem cell migration and mediating cell survival in response to ischemic injury^20^. Recently, SDF-1α expression has been found to increase after acute myocardial infarction, and its chemotactic properties have been demonstrated to confer cardiac protection and promote regeneration^21^,^22^,^23^. However, native SDF-1α diffuses quickly *in vivo*, which will reduce its local concentration and therapeutic efficiency. Many studies have reported that delivery of SDF-1α gene on DNA plasmid or viral vector had some effect in cardiac repair. However, the introduction of ectopic transgenes limits their current therapeutic application. Gene-based methods have been hampered by poor control of dosage and duration, low gene-transfer efficiency, risk of genomic integration and associated tumorigenesis, and antiviral immune responses^24^,^25^. Zangi *et al.* reported that VEGF-A DNA plasmid-treated hearts displayed obvious edema and vessels showed excessive permeability, which was likely due to prolonged exposure to VEGF-A with DNA-mediated gene transfer, as increased vascular permeability is a known consequence of lengthy VEGF-A expression^26^.

Many studies have focused on how to maintain the local concentration by using multiple injections, but this strategy increases the cost and produces adverse effects. Thus, it is important to identify a suitable delivery system for the controlled release of SDF-1α. Type I collagen is the main component of the cardiac extracellular matrix, and its expression is increased in the ischemic area after acute myocardial infarction^17^,^27^. Therefore, type I collagen may be a potential target for the controlled release of SDF-1α in the infarcted heart. In the present study, we incorporated the collagen-binding domain into SDF-1α to produce a collagen-binding SDF-1α (CBD-SDF-1α). Because the active site of native SDF-1α is at the N-terminus, we fused the collagen-binding domain to the C-terminus of the protein and separated the two proteins with a linker. Our results demonstrate that CBD-SDF-1α has similar chemotactic activity as native SDF-1α in inducing MSC or HSC migration, which indicates that the bioactivity of SDF-1α is not affected by incorporating the collagen-binding domain. Using *in vitro* assays, we demonstrated that CBD-SDF-1α could specifically bind to a collagen gel and achieve controlled release. Moreover, a CBD-SDF-1α-modified collagen gel was conducive to stem cell adhesion.

Using an acute myocardial infarction model, we injected the chemokine into the ischemic area and showed that the CBD-SDF-1α was tethered in the ischemic area and diffused slowly. These results indicate that CBD-SDF-1α can also bind to endogenous collagen and achieve controlled release *in vivo*. This delivery system helped to enhance the local concentration and prolong the therapeutic efficiency in the ischemic area.

Previous studies have reported that adult c-kit^+^ stem cells in the epicardium are necessary for cardiac regeneration and repair^28^,^29^,^30^. These endogenous c-kit^+^ stem cells may replenish the dysfunctional cardiomyocytes, promote angiogenesis and ultimately improve cardiac function. In the present study, improvements in cardiac function were also accompanied by the increased recruitment of c-kit^+^ stem cells and increased capillary density in the ischemic area. We have also detected other types of stem cells in the ischemic area using their specific markers, such as CD34 for hematopoietic stem cell (HSCs) and CD31 for endothelial progenitor cells (EPCs). However, CD34 and CD31 positive cells have not been found in the ischemic area, which may due to small quantity of them in the peripheral blood (data not shown).

Revascularization is very important for cell survival, differentiation and cardiac regeneration^31^,^32^. The recruitment of endogenous stem cells may promote the secretion of many nutrient factors that promote angiogenesis and cell proliferation. Many studies have found that the recruited stem cells can secret some trophic factors to promote cardiac repair, while VEGF is a key therapeutic trophic factor^19^,^24^,^28^. Therefore, we detected the VEGF in the ischemic area at day 4 post-surgery. The results of western blot showed that CBD-SDF-1α group had much higher VEGF concentration than other two groups, and the elevation of VEGF in CBD-SDF-1α group was due to its higher efficiency in the recruitment of stem cells. SDF-1α itself can also promote angiogenesis^33^,^34^. Our results also demonstrate that the recruitment of stem cells was accompanied by a reduced scar size and increased LV wall thickness, suggesting the greater advantages of using CBD-SDF-1α for cardiac repair.

Zangi *et al.* reported that controlled release of VEGF-A via VEGF-A modified mRNA is a novel strategy for mobilizing endogenous heart progenitors, which gives us some guiding advices for our research. modRNA is an effective, robust approach to implement to delivery protein in damaged tissues, which avoids several of the apparent problems that have arisen with conventional cardiac gene therapy vectors, including lack of genomic integration, persistence of expression, immunogenicity, need for life-long monitoring for tumorigenesis and other adverse clinical outcomes^24^. In the present study, the CBD fused proteins provide an alternative approach for sustained release of proteins locally. On one hand, CBD-SDF-1α can be directly injected in the ischemic area, where is rich of collagen. On the other hand, CBD-SDF-1α can be readily immobilized on the collagen scaffold to construct a smart biomaterial for tissue regeneration, such as functionalized cardiac patch. Therefore, our approach has more extensive applications in tissue regeneration.

In summary, although our study is limited by the use of a single molecule, SDF-1α, the findings demonstrate that suitable delivery of this protein is sufficient to induce endogenous stem cell homing to the injured heart and promote cardiac repair. Moreover, we have described a potential delivery strategy for directing stem cell infiltration into the injured tissues that may also have general applications in the regeneration of other tissues and open a new avenue for the controlled delivery of other proteins to the injured sites.

## Methods

### Cloning, expression and purification of SDF-1α

The native human SDF-1α (NAT-SDF-1α) gene was amplified from human fibroblast cDNA by polymerase chain reaction. CBD-SDF-1α was constructed by incorporating the collagen-binding domain (CBD) into the C-terminus of NAT-SDF-1α. The gene sequence of SDF-1α was separated from the CBD sequence to preserve its activity by a linker (amino acid sequence: GSAGSAAGSGG). NAT-SDF-1α or CBD-SDF-1α was inserted into the pET28a vector (Novagen, Madison, WI), and then transformed into the BL21 strain (DE3) of *Escherichia coli* (Novagen, Madison, WI) for protein expression. Both proteins containing a 6 × histidine tag were purified by a Nickel affinity chromatography column (GE Healthcare) and identified by Tricine SDS-PAGE and western blotting using an anti-polyhistidine monoclonal antibody (1:3000; Sigma-Aldrich, St. Louis, MO, USA).

### Collagen-binding and controlled release experiments *in vitro*

The collagen-binding assay was performed using a modified ELISA assay. The collagen solution was prepared from rat tail tendon as previously described and added to a 96-well plate. The plate was incubated at 4 °C overnight and then dried after discarding the solution. After washing and blocking, increasing concentrations of an NAT-SDF-1α or CBD-SDF-1α solution were added to the plate (100 μL/well), incubated at 37 °C for 2 hours, and then washed 3 times to remove the unbound proteins. The proteins bound to collagen were detected by a primary antibody against polyhistidine and an alkaline phosphatase-conjugated secondary antibody (1:10,000; Sigma-Aldrich, St. Louis, MO, USA). The OD values were quantified at 405 nm using an ELISA reader (Molecular Devices), and the dissociation constants (*K*_d_), of NAT-SDF-1α and CBD-SDF-1α from collagen were calculated by Scatchard analysis.

In the release assay, the chemokines were bound to the collagen-coated 96-well plate in advance as described above, and 200 μL of PBS was added to each well. The plate was incubated on a rocker platform (37 °C, 80 g) to simulate body fluid erosion, and the samples were collected and replaced with PBS every 24 hours. At each time point from day 0–8, the concentration of SDF-1α in the samples was analyzed with a human SDF-1α ELISA kit according to the manufacturer’s protocol (Bluegene, Shanghai, China).

### Chemotactic activity of NAT-SDF-1α and CBD-SDF-1α *in vitro*

Mouse hematopoietic stem cells (mHSCs) were freshly isolated from the bone marrow of 4-week-old male *C57BL/6J* mice by a magnetic activated cell sorting kit (BD Biosciences), and human placenta-derived mesenchymal stem cells (hMSCs) were isolated from neonatal placenta from healthy volunteers as previously described^31^. The identity of HSCs was confirmed by co-staining of CD117 and DAPI. For MSCs, due to no unique marker to identify them, we applied a typical standard to identify them by flow cytometry and the differentiation capacity into osteoblasts, adipocytes, and chondrocytes was also studied.

A modified Boyden chamber (Corning Costar, 8μm) assay was performed to test the chemotactic activity of NAT-SDF-1α and CBD-SDF-1α. Briefly, 600 μL of NAT-SDF-1α, CBD-SDF-1α (100 ng/mL) or medium alone was added to the lower chamber and 200 μL of mHSCs or hMSCs was seeded into the upper chamber at a density of 2 × 10^4^ cells/mL. After incubation at 37 °C for 12 hours, the samples were fixed and stained with crystal violet. The mean numbers of migrated cells from five fields were observed and counted under a microscope (200×). The migration index was calculated to express the stimulated migration using the following equation: Migration index = Stimulated migration/Random migration.

### mHSC and hMSC adhesion assay

The adhesion assay was performed to detect the adhesive ability of the mHSCs and hMSCs mediated by NAT-SDF-1α or CBD-SDF-1α. Briefly, a chemokine-modified collagen gel was prepared as the binding substrate in a 24-well plate. The mHSCs and hMSCs were seeded into each well (2 × 10^4^/well), and the medium was discarded after incubation at 37 °C for 1 hour. After gentle washing with PBS, the retained cells were fixed with 4% paraformaldehyde, and mHSCs were co-stained with anti-CD117 and DAPI. For MSCs, due to no unique marker, the retained cells were stained with DAPI only. Six fields of view were randomly imaged using a Zeiss Z1 fluorescent microscope, and the Hoechst-positive cells were counted.

### Acute myocardial infarction model and recombinant chemokine injection

Male Sprague-Dawley (SD) rats weighing 180 g to 200 g were used for the acute myocardial infarction (AMI) model. Briefly, the rats were anesthetized with sodium pentobarbital (40 mg/kg), intubated and ventilated with room air. After a thoracotomy was performed at the left fourth intercostal space, the left anterior descending coronary artery (LAD) was permanently ligated with a 6–0 silk suture below the tip of the left atrial appendage. Immediately after ligating the left anterior descending coronary artery, 1.0 nmol of NAT-SDF-1α or CBD-SDF-1α dissolved in 100 μL of PBS was injected into the infarcted border zone at 5 sites. After the injection, routine chest closure was performed and the rats were allowed to recover on a heating pad.

### CBD-SDF-1α binding ability in the infarcted heart

To detect the retained SDF-1α at the border zone of the ischemic area, the hearts were harvested at 3 hours, 6 hours, 4 days and 7 days after the injection. The peri-infarct zones were dissected and immediately frozen in liquid nitrogen for protein extraction. Western blotting with a primary antibody against polyhistidine was used to distinguish the exogenous SDF-1α from the endogenous protein. In addition, the concentration of SDF-1α in the serum at each time point was detected by using the human SDF-1αELISA kit.

### Measurement of the c-kit^+^ stem cells and VEGF in the ischemic myocardium

Immunofluorescence staining was used to identify endogenous stem cells homing to the ischemic area. The hearts were harvested at 4 days after surgery and embedded in Tissue-Tek optimal cutting temperature (O.C.T.) compound (Sakura, USA). Frozen sections (7 μm) were prepared and incubated overnight with the primary antibody against c-kit (1:200, Abcam) at 4 °C. After washing, the sections were incubated with the FITC-conjugated donkey anti-mouse IgG (1:500, Abcam) at room temperature for 2 hours and the nuclei were counterstained with DAPI. Six fields of each sample were randomly imaged by confocal microscopy and measured by an observer blind to the treatment group with Image-pro Plus software (Media Cybernetics, Version 6.0). We also detected the expression of VEGF in the ischemic area by western blot, because VEGF is a very important cytokine for cardiac regeneration in the ischemic heart.

### Analysis of cardiac function by echocardiography

At 12 weeks after surgery, echocardiography was performed to assess cardiac function using a 10-MHz linear transducer and a cardiovascular ultrasound system (SONOS model 5500, Hewlett-Packard, Palo Alto, CA). The left ventricular (LV) end-systolic dimension (LVDs) and end-diastolic dimension (LVDd) were measured in M-mode tracings at the midpapillary level. The LV end-diastolic volume (LVEDV), the LV end-systolic volume (LVESV), the ejection fraction (EF) and fractional shortening (FS) were measured to examine the systolic function.

### Histological analysis

All hearts were harvested after the echocardiographic measurements and fixed in 4% paraformaldehyde. The samples were embedded in paraffin, and 4 μm-thick sections were cut and mounted on positively charged glass slides. Masson’s trichrome-staining was used to analyze the scar size and the thickness of the left ventricle (LV) wall. An anti-von Willebrand Factor (vWF) antibody (1:500, Abcam, USA) was used to evaluate the density of the capillary vessels. Image-Pro Plus software was used to quantify the capillary density.

### Statistical analysis

Statistical analysis was performed with SPSS soft ware for Windows (version 13.0, SPSS Inc., Chicago, IL, USA). Comparisons among multiple groups were performed by one-way analyses of variance (ANOVA) followed by the Bonferroni-Dunn test. Comparisons between two groups were performed using the unpaired Student t-test. All data are expressed as the mean ± SEM; a value of *P* < 0.05 was considered to represent a significant difference.

### Study approval

All animal procedures were approved by the Third Military Medical University Animal Care Committee and in accordance with the National Institute of Health’s Guide for the Care and Use of Laboratory Animals.

## Additional Information

**How to cite this article**: Sun, J. *et al.* Controlled Release of Collagen-Binding SDF-1α Improves Cardiac Function after Myocardial Infarction by Recruiting Endogenous Stem Cells. *Sci. Rep.* **6**, 26683; doi: 10.1038/srep26683 (2016).

## Supplementary Material

Supplementary Information

## Figures and Tables

**Table 1:** Evaluation of Cardiac Function by Echocardiography (12 Weeks after Injection).

Groups	LVDd, cm	LVDs, cm	IVSDT, cm	LVPWT, cm	EF, %	FS, %
Control	0.73 ± 0.026	0.45 ± 0.106	0.13 ± 0.034	0.22 ± 0.005	42.54 ± 2.1	18.35 ± 1.4
NAT-SDF-1α	0.71 ± 0.013	0.51 ± 0.037	0.15 ± 0.016	0.23 ± 0.007	57.69 ± 1.8	26.85 ± 2.3
CBD-SDF-1α	0.69 ± 0.041	0.40 ± 0.029	0.14 ± 0.028	0.25 ± 0.019	72.14 ± 1.6*	36.36 ± 1.5*

FS, fractional shortening; EF, ejection fraction; LVDd, left ventricular end-diastolic dimension; LVDs, left ventricular end-systolic dimension; IVSDT, interventricular septal diastolic thickness; and LVPWT, left ventricular posterior wall thickness. The data are the means ± SD, n = 8 in each group. *P < 0.05 *vs.* the controls.

## References

[b1] Tomita S. *et al.* Autologous Transplantation of Bone Marrow Cells Improves Damaged Heart Function. Circulation 100, 247–256 (1999). 10.1161/01.cir.100.suppl_2.ii-247 10567312

[b2] Macia E. & Boyden P. A. Stem cell therapy is proarrhythmic. Circulation 119, 1814–1823 (2009). 19349334 10.1161/CIRCULATIONAHA.108.779900 PMC2739413

[b3] Atluri P. *et al.* Acute myocardial rescue with endogenous endothelial progenitor cell therapy. Heart Lung Circ 19, 644–654 (2010). 20719564 10.1016/j.hlc.2010.06.1056 PMC3235678

[b4] Wang Y., Haider H., Ahmad N., Zhang D. & Ashraf M. Evidence for ischemia induced host-derived bone marrow cell mobilization into cardiac allografts. J Mol Cell Cardiol 41, 478–487 (2006). 16926022 10.1016/j.yjmcc.2006.06.074

[b5] Bollini S., Smart N. & Riley P. R. Resident cardiac progenitor cells: at the heart of regeneration. J Mol Cell Cardiol 50, 296–303 (2011). 20643135 10.1016/j.yjmcc.2010.07.006

[b6] Pourrajab F., Babaei Zarch M., Baghi Yazdi M., Rahimi Zarchi A. & Vakili Zarch A. Application of stem cell/growth factor system, as a multimodal therapy approach in regenerative medicine to improve cell therapy yields. Int J Cardiol 173, 12–19, (2014). 24612559 10.1016/j.ijcard.2014.02.006

[b7] Ellison G. M., Nadal-Ginard B. & Torella D. Optimizing Cardiac Repair and Regeneration Through Activation of the Endogenous Cardiac Stem Cell Compartment. J Cardiovasc Transl Res 5, 667–677 (2012). 22688972 10.1007/s12265-012-9384-5

[b8] Smart N. & Riley P. R. The stem cell movement. Circ Res 102, 1155–1168 (2008). 18497316 10.1161/CIRCRESAHA.108.175158

[b9] Ziegler M. *et al.* The bispecific SDF1-GPVI fusion protein preserves myocardial function after transient ischemia in mice. Circulation 125, 685–696 (2012). 22223428 10.1161/CIRCULATIONAHA.111.070508

[b10] Ghadge S. K., Muhlstedt S., Ozcelik C. & Bader M. SDF-1alpha as a therapeutic stem cell homing factor in myocardial infarction. Pharmacol Ther 129, 97–108 (2011). 20965212 10.1016/j.pharmthera.2010.09.011

[b11] Saxena A. *et al.* Stromal Cell-Derived Factor-1 Is Cardioprotective After Myocardial Infarction. Circulation 117, 2224–2231 (2008). 18427137 10.1161/CIRCULATIONAHA.107.694992 PMC2743260

[b12] Hu X. *et al.* Stromal cell derived factor-1 alpha confers protection against myocardial ischemia/reperfusion injury: role of the cardiac stromal cell derived factor-1 alpha CXCR4 axis. Circulation 116, 654–663 (2007). 17646584 10.1161/CIRCULATIONAHA.106.672451 PMC3640445

[b13] Takahashi M. Role of the SDF-1/CXCR4 System in Myocardial Infarction. Circ. J 74, 418–423 (2010). 20118565 10.1253/circj.cj-09-1021

[b14] Zhang M. *et al.* SDF-1 expression by mesenchymal stem cells results in trophic support of cardiac myocytes after myocardial infarction. FASEB J 21, 3197–3207 (2007). 17496162 10.1096/fj.06-6558com

[b15] Blumenthal B. *et al.* Functional regeneration of ischemic myocardium by transplanted cells overexpressing stromal cell-derived factor-1 (SDF-1): intramyocardial injection versus scaffold-based application. Eur J Cardiothorac Surg 40, e135–141 (2011). 21684755 10.1016/j.ejcts.2011.05.026

[b16] Purcell B. P., Elser J. A., Mu A., Margulies K. B. & Burdick J. A. Synergistic effects of SDF-1alpha chemokine and hyaluronic acid release from degradable hydrogels on directing bone marrow derived cell homing to the myocardium. Biomaterials 33, 7849–7857 (2012). 22835643 10.1016/j.biomaterials.2012.07.005 PMC3449064

[b17] Cleutjens J. P., Verluyten M. J., Smiths J. F. & Daemen M. J. Collagen remodeling after myocardial infarction in the rat heart. Am J Pathol 147, 325–338 (1995). 7639329 PMC1869816

[b18] de Souza S. J. & Brentani R. Collagen Binding Site in Collagenase can be Determined Using the concept of sense-antisense peptide interactions. J Biol Chem 267, 13763–13767 (1992). 1320031

[b19] Zhang J. *et al.* Collagen-targeting vascular endothelial growth factor improves cardiac performance after myocardial infarction. Circulation 119, 1776–1784 (2009). 19307480 10.1161/CIRCULATIONAHA.108.800565

[b20] Nilsson J., Ali S., Harvey I., Kirby J. A. & Meeson A. P. Stem cell therapy: a role for CXCR4 in homing bone marrow side population cells to areas of myocardial damage. Int J Cardiol 145, 554–555 (2010). 20557955 10.1016/j.ijcard.2010.05.021

[b21] Askari A. T. *et al.* Effect of stromal-cell-derived factor 1 on stem-cell homing and tissue regeneration in ischaemic cardiomyopathy. The Lancet 362, 697–703 (2003). 10.1016/S0140-6736(03)14232-8 12957092

[b22] Fraidenraich D. *et al.* Rescue of cardiac defects in id knockout embryos by injection of embryonic stem cells. Science 306, 247–252 (2004). 15472070 10.1126/science.1102612 PMC1351017

[b23] Abbott J. D. *et al.* Stromal cell-derived factor-1alpha plays a critical role in stem cell recruitment to the heart after myocardial infarction but is not sufficient to induce homing in the absence of injury. Circulation 110, 3300–3305 (2004). 15533866 10.1161/01.CIR.0000147780.30124.CF

[b24] Zangi L. *et al.* Modified mRNA directs the fate of heart progenitor cells and induces vascular regeneration after myocardial infarction. Nat Biotechno l31, 898–907(2013). 10.1038/nbt.2682 PMC4058317 24013197

[b25] Spilsbury K., Garrett K. L., Shen W. Y., Constable I. J. & Rakoczy P. E. Overexpression of vascular endothelial growth factor (VEGF) in the retinal pigment epithelium leads to the development of choroidal neovascularization. Am. J. Pathol 157, 135–144 (2000). 10880384 10.1016/S0002-9440(10)64525-7 PMC1850220

[b26] Nagy J. A. *et al.* Permeability properties of tumor surrogate blood vessels induced by VEGF-A. Lab. Invest 86, 767–780 (2006). 16732297 10.1038/labinvest.3700436

[b27] Jugdutt B. I. Ventricular remodeling after infarction and the extracellular collagen matrix: when is enough enough? Circulation 108, 1395–1403 (2003). 12975244 10.1161/01.CIR.0000085658.98621.49

[b28] Zisa D., Shabbir A., Suzuki G. & Lee T. Vascular endothelial growth factor (VEGF) as a key therapeutic trophic factor in bone marrow mesenchymal stem cell-mediated cardiac repair. Biochem Biophys Res Commun 390, 834–838 (2009). 19836359 10.1016/j.bbrc.2009.10.058 PMC2788008

[b29] Gnecchi M., Zhang Z., Ni A. & Dzau V. J. Paracrine mechanisms in adult stem cell signaling and therapy. Circ Res 103, 1204–1219 (2008). 19028920 10.1161/CIRCRESAHA.108.176826 PMC2667788

[b30] Ellison G. M. *et al.* Adult c-kit(pos) cardiac stem cells are necessary and sufficient for functional cardiac regeneration and repair. Cell 154, 827–842 (2013). 23953114 10.1016/j.cell.2013.07.039

[b31] Proulx C. *et al.* Antagonism of stromal cell-derived factor-1alpha reduces infarct size and improves ventricular function after myocardial infarction. Pflugers Arch 455, 241–250 (2007). 17520275 10.1007/s00424-007-0284-5

[b32] Segret A. *et al.* Structural localization and expression of CXCL12 and CXCR4 in rat heart and isolated cardiac myocytes. J Histochem Cytochem 55, 141–150 (2007). 17046839 10.1369/jhc.6A7050.2006

[b33] Segers V. F. *et al.* Local delivery of protease-resistant stromal cell derived factor-1 for stem cell recruitment after myocardial infarction. Circulation 116, 1683–1692 (2007). 17875967 10.1161/CIRCULATIONAHA.107.718718

[b34] Wang Y. *et al.* Effects of hypoxia on osteogenic differentiation of rat bone marrow mesenchymal stem cells. Mol Cell Biochem 362, 25–33 (2012). 22198287 10.1007/s11010-011-1124-7

