# Supplementary material for: Controlled Release of Collagen-Binding SDF-1α Improves Cardiac Function after Myocardial Infarction by Recruiting Endogenous Stem Cells
Source: Sci Rep. 2016 May 26;6:26683. doi: 10.1038/srep26683 (PMC4881239; doi:10.1038/srep26683)
Supplement: Supplementary Information [file srep26683-s1.pdf]

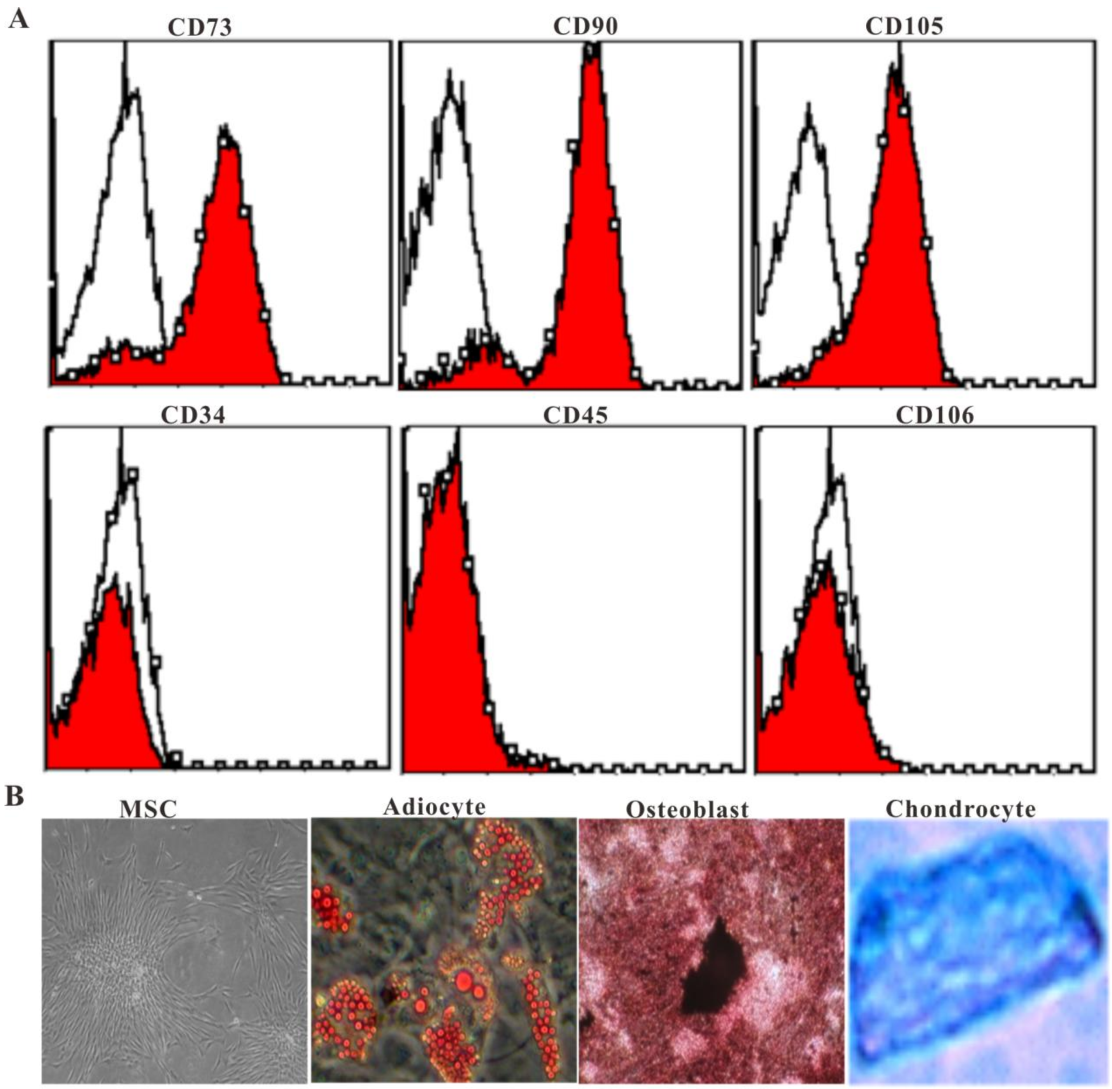

Figure S1: Identification of hMSC. (A) The results of flow cytometry showed that MSCs are positive for CD73, CD90 and CD105, while negative for CD34, CD44 and CD106. (B) MSCs have uniform fibroblast-like morphology and can differentiate into adiocytes, osteoblasts and chondrocytes.
